# Supplementary material for: Developing the Leuven Embedded Figures Test (L-EFT): testing the stimulus features that influence embedding
Source: PeerJ. 2017 Jan 24;5:e2862. doi: 10.7717/peerj.2862 (PMC5267572; doi:10.7717/peerj.2862)
Supplement: Supplemental Information 1 [file peerj-05-2862-s003.pdf]

Data Set Experiment 1

Variables	Description
TrialNumber	The number of the trial presented to the participant (ranging from 0 to 63).
N_target	The number of lines of which the target is made up (ranging from 3 to 8).
N_cont	The number of lines continued from the target into the embedding context (ranging from 0 to a maximum equal to the number of target lines).
N_alter	The number of lines in the two embedding contexts that did not contain the target (distractors).
N_crossing	The number of lines across the target shape in the embedding context.
Closure	Is the target shape open (0) or closed (1)?
Symmetry	Is the target shape symmetric (1) or asymmetric (0)?
Acc	Was the first response made by the participant accurate? 0 if participant chose one of the two wrong response alternatives, and 1 if he chose the correct response alternative.
NumberofResponses	How many responses did the participant make before answering correct?
participant.ID	The unique user ID of the participant
RT	The response time of the participant for the first response they made (in milliseconds).

Data Set Experiment 2

N_target	The number of lines of which the target is made up (ranging from 3 to 8).
N_cont	The number of lines continued from the target into the embedding context (ranging from 0 to a maximum equal to the number of target lines).
N_alter	The number of lines in the two embedding contexts that did not contain the target (distractors).
N_crossing	The number of lines across the target shape in the embedding context.
Closure	Is the target shape open (0) or closed (1)?
Symmetry	Is the target shape symmetric (1) or asymmetric (0)?
participant.ID	The unique user ID of the participant

TrialNumber	The number of the trial presented to the participant (ranging from 1 to 128).
run	The block of trials. 0 for the first block of 64 trials and 1 for the second block of 64 trials.
RT	The response time of the participant (in seconds).
Acc	Was the response of the participant correct? 0 if participant chose incorrect alternative, and 1 if they chose correct alternative.
